# Liver‐specific lncRNAs associated with liver cancers

**DOI:** 10.1002/2211-5463.70079

**Published:** 2025-07-01

**Authors:** Olga Y. Burenina, Roman K. Makarchenko, Maria P. Rubtsova, Olga A. Dontsova

**Affiliations:** ^1^ Center of Molecular and Cellular Biology Moscow Russia; ^2^ Chemistry Department Lomonosov Moscow State University Russia; ^3^ Shemyakin–Ovchinnikov Institute of Bioorganic Chemistry, Russian Academy of Sciences Moscow Russia

**Keywords:** biomarkers, hepatocellular carcinoma, liver cancer, long noncoding RNA

## Abstract

Long non‐coding RNAs (lncRNAs) are transcripts with a length more than 200 nt, which do not encode proteins and act just as RNA molecules. In general, lncRNAs have much more distinct tissue specificity than proteins, as they usually realize more peculiar regulatory functions. Their expression levels are often altered in a response to stress conditions, metabolic changes, development of different diseases, and carcinogenesis. Cancer‐associated lncRNAs are widely considered as perspective and useful biomarkers. Thus, development of clinical tests, which include tissue‐specific and cancer‐specific lncRNAs, might significantly contribute to cancer diagnostics and/or prognosis of the disease. A number of lncRNAs is known to be dysregulated in liver tumors and considered as probable biomarkers. However, most of them are rather universally well‐known lncRNAs associated with various cancers. In the present review, we aimed to shed light on other lncRNAs with preferential expression in liver and/or liver tumors, for example, *LINC01554*, *LINC01093*, *LINC01348, LINC02428*, *FAM99B*, *etc*. We summarized recent discoveries unveiling their dysregulation in liver malignancies and related cellular mechanisms in which they are involved and considered their significance as probable liver cancer biomarkers.

## Graphical abstract

Long non‐coding RNAs (lncRNAs) are regulatory molecules with various functions. They are more tissue‐specific than proteins and can be used as potential biomarkers, particularly in cancer diagnostics and prognosis. In this review, we have systematically compiled all lncRNAs with exclusive expression in the human liver, verified their liver specificity in available databases, and discussed their role in liver cancer.

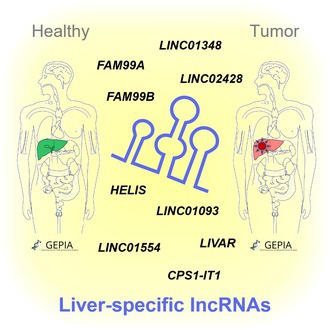

**Abbreviations**
AFP: alpha‐fetoprotein
CCA: cholangiocarcinoma
ceRNA: competitive endogenous RNA
EMT: epithelial–mesenchymal transition
HBL: hepatoblastoma
HBV: hepatitis B virus
HCC: hepatocellular carcinoma
HCV: hepatitis C virus
lncRNA: long noncoding RNA
NAFLD: nonalcoholic fatty liver disease
NASH: nonalcoholic steatohepatitis
nt: nucleotides
RIP: RNA immunoprecipitation
TCGA: The Cancer Genome Atlas

LncRNAs comprise a heterogeneous class of transcripts with a length of more than 200 nt, which do not undergo translation. They act as regulatory RNA molecules through various mechanisms, including protein scaffolding, sponging microRNAs, interacting with mRNAs or other lncRNAs, or via direct binding to DNA promoters [1, 2]. Being a powerful tool for the regulation of gene expression, lncRNAs are largely involved in a number of key cellular processes, in particular those mechanisms specific to various pathogenic conditions and carcinogenesis [3, 4, 5]. Thus, lncRNAs are widely considered in terms of cancer therapy and diagnostics [6]. Besides, some of them are secreted in blood and might be used as noninvasive oncomarkers [7]. So far, only a single lncRNA – *PCA3* is already used in routine clinical tests as an efficient oncomarker for prostate cancer, which has exclusive tissue specificity and cancer specificity and can be directly detected in urine samples [8]. Indeed, tissue specificity is more pronounced for lncRNAs than for proteins according to RNA‐Seq data, even considering only normal human tissues [9, 10, 11]. Additionally, different types of tumors express different sets of lncRNAs, and at least some novel candidates can appear to be unique to distinct malignancies; thus, they are a powerful tool for their specific diagnostics and potential targets for cancer therapy [12, 13].

Up to date hundreds of lncRNAs associated with liver tumors are investigated and not once described in the literature [14, 15, 16, 17]. Mainly these reviews are focused on lncRNAs dysregulated in hepatocellular carcinoma (HCC), whereas some studies are devoted to cholangiocarcinoma (CCA) specifically [18, 19]. Regardless of the exact type of tumor the most well‐known lncRNAs in terms of liver cancer are universal oncogenic molecules, such as *MALAT1*, *HOTTIP*, *HOTAIR*, *NEAT1*, *DANCR*, *CYTOR*, *H19*, *MEG3*. Some of these lncRNAs can be found in blood exosomes as circulating biomarkers [20] enhancing their common diagnostic potential. However, none of them are liver‐specific or HCC‐specific, as they are dysregulated in the majority of malignancies, that diminishes their significance for specialized liver cancer diagnostics [21].

One of the most well‐known liver‐specific cancer‐associated lncRNA is *HULC*, that was firstly identified to be overexpressed in HCC and thus named as “Highly Upregulated in Liver Cancer” [22]. *HULC* can be also secreted into blood of HCC patients and its high concentration was associated with high *HULC* expression in cancer tissues [23]. Functional role of *HULC* is widely investigated [24, 25, 26, 27]. However, recent studies discovered a number of other liver‐specific lncRNAs, which are not so well‐studied as *HULC* yet. They also have high diagnostic/prognostic potential for liver cancers, and current investigations are focused on revealing their functional role in carcinogenesis and possible applications. In the present review we for the first time summarized data about known lncRNAs with the pronounced expression predominantly in liver tissues. Some of them are already investigated to some extent, for example, *LINC01554*, *LINC01093*, *LINC01348, LINC02428, FAM99B*. For others, there are only few scattered evidences of their probable functions or just single publications. With the help of this study, we aim to uncover these novel lncRNAs candidates for future investigations. However, some of lncRNAs firstly discovered as liver‐specific turned out to be expressed in other tissues and/or other tumors as well. To clarify these issues, we performed additional analysis and collected expression profiles of all lncRNAs mentioned in this review. We present these data as illustrative heatmaps, firstly containing information for healthy human tissues (Fig. 1) downloaded from GTEx portal (www.gtexportal.org, [28]). Next, we explored expression levels of described lncRNAs in The Cancer Genome Atlas (TCGA, portal.gdc.cancer.gov, [29]) datasets, visualized by GEPIA browser (Gene Expression Profiling Interactive Analysis, gepia.cancer‐pku.cn, [30]). We provided in a Supplementary corresponding boxplots for lncRNA expression in HCC and CCA compared to adjacent normal liver tissues (Fig. S1), as well as survival plots (Kaplan–Meier curves for overall survival, Fig. S2) to show probable prognostic value of those lncRNAs. Considering insufficient expression of most of the described lncRNAs in CCA and small sample size (*n* = 36) in TCGA dataset we showed survival plots only for HCC patients (Fig. S2). Additionally, we compared expression levels of all described lncRNAs across different tumors using TCGA data in a view of pan‐cancer matrix plot (Fig. S3). As far as most of the lncRNAs turned out to be downregulated in HCC and CCA, it shows mainly some low background expression of these lncRNAs. To explore whether dysregulation of lncRNA expression levels is a feature of liver carcinogenesis or it takes place in other liver diseases as well, we used the data available in the GepLiver atlas (www.gepliver.org, [31]) and presented them as a corresponding heatmap (Fig. 2). Therefore, we provided a comprehensive description of the all liver‐specific lncRNAs mentioned in the literature to date, and combined it with a verification of these data in current databases.

**Fig. 1:**
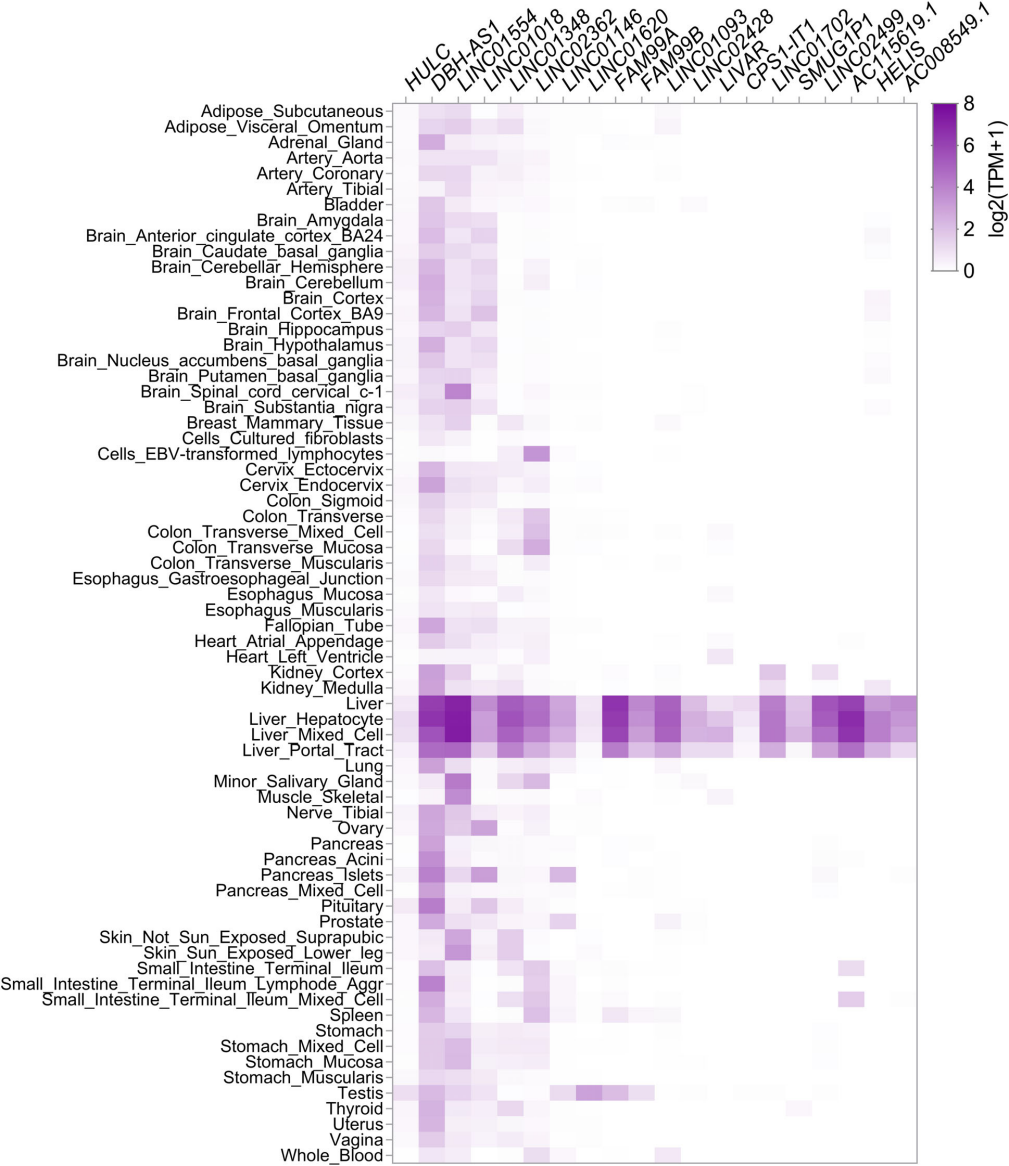
Median gene‐level expression [log_2_(TPM+1)] of described lncRNAs in different human tissues/cell types according to the open access dataset (GTEx_Analysis_v10_RNASeQCv2.4.2_gene _median_tpm.gct.gz) downloaded from GTEx Portal (www.gtexportal.org, [28]).

**Fig. 2:**
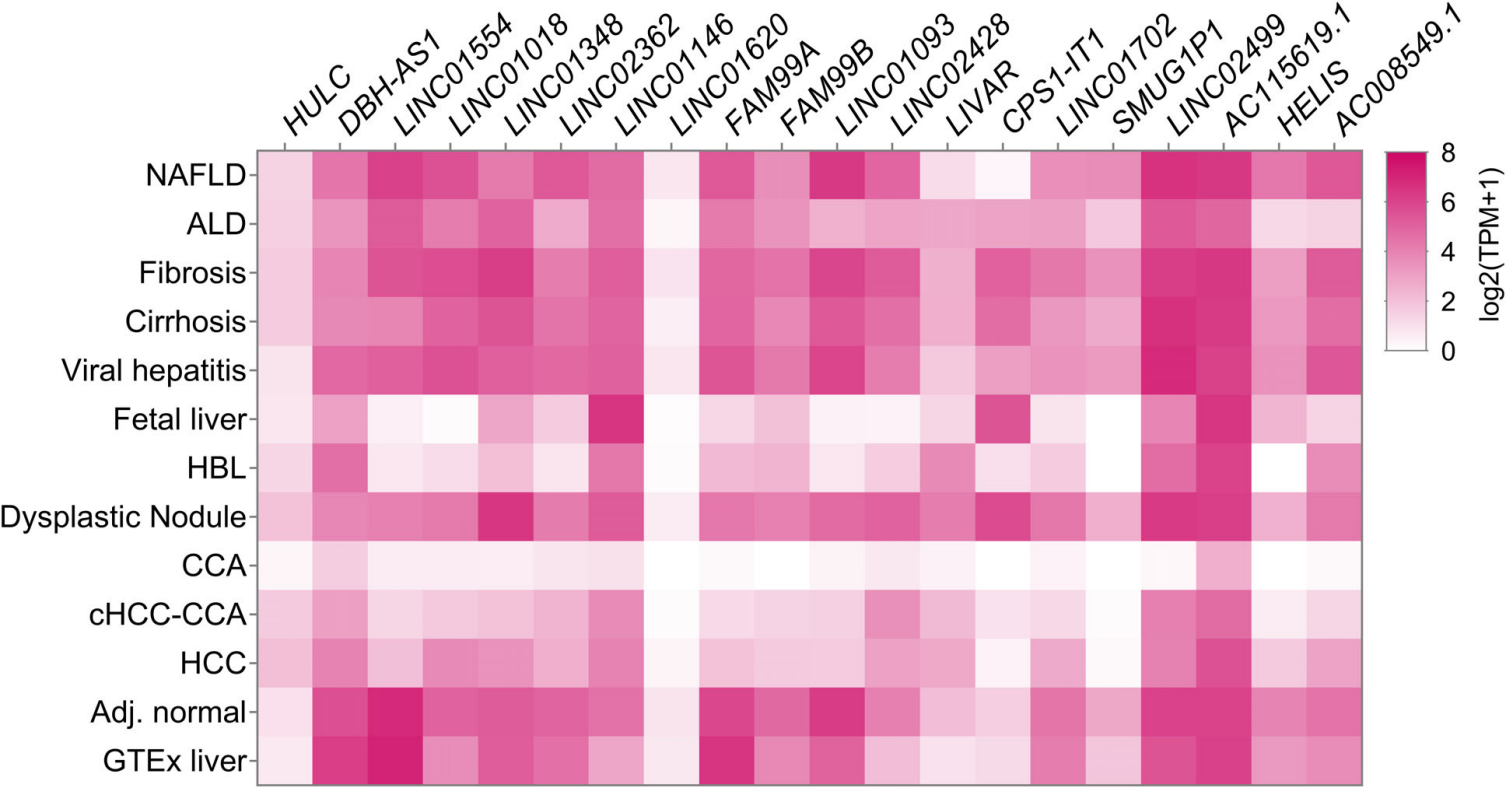
Median gene‐level expression [log_2_(TPM+1)] of described lncRNAs in different liver diseases and conditions in comparison to normal GTEx data and bulk adjacent liver tissues (Adj. normal) according to the open access dataset (Hsa_mRNA_lncRNA_anno.txt) downloaded from GepLiver atlas (http://www.gepliver.org/#/download, [31]). Abbreviations: ALD, alcoholic liver disease; CCA, cholangiocarcinoma; HBL, hepatoblastoma; HCC, hepatocellular carcinoma; NAFLD, nonalcoholic fatty liver disease; сHCC‐CCA: combined HCC‐CCA.

## Liver‐specific gene expression

Although all human tissues function in a view of common fundamental processes, tissues are distinguished by specific gene expression patterns that determine their identity and imply distinct regulatory programs. The phenomenon of liver‐specific gene expression has been known for a long time, as the liver is involved in a great variety of peculiar physiological and metabolic processes, turnover of blood toxins, control of lipids, along with many other functions [32]. The liver consists of multiple cell types, including endothelial and stellate cells, cholangiocytes, and Kupffer cells; however, up to 60% of the cell population and up to 80% of liver volume are accounted for by hepatocytes, which are responsible for the production of albumin, fibrinogen, transferrin, glycoproteins, lipoproteins, etc., [33]. Specific activation of gene expression is largely maintained by well‐known liver‐enriched transcription factors such as different hepatocyte nuclear factors (HNF), including key players HNF1α, HNF4α, HNF6, and FOXA2 (reviewed in [34, 35]). Probably the most well‐known liver‐specific protein is the membrane asialoglycoprotein receptor ASGPR, widely used for highly efficient target delivery of various molecules and particles conjugated with N‐acetylgalactosamine (GalNAc) [36, 37]. Recent proteomics and transcriptomics studies allowed for revealing not only full protein repertoires inherent in liver development, metabolism, and disease but also a large scale of noncoding transcripts [38]. Many researches are focused on panels of lncRNAs dysregulated in liver pathologies such as nonalcoholic fatty liver disease (NAFLD), nonalcoholic steatohepatitis (NASH), fibrosis, and cirrhosis [39, 40, 41], as well as considering validation of *in vitro* liver cell models [42]. However, the hottest topic is the deregulation of gene expression upon liver carcinogenesis.

## Liver carcinogenesis

Primary liver cancer is the sixth most common malignancy and fourth leading cause of mortality from cancer worldwide [43, 44]. HCC is the predominant liver cancer, accounting for 75–85% of all cases, followed by intrahepatic CCA [45, 46]. Although novel therapeutic approaches largely increase patient survival rates [47, 48], both types of tumors are usually asymptomatic at early stages, which highly compromises therapeutic options [46, 49]. HCC and CCA originate, correspondingly, from hepatocytes and bile duct cells, whereas a mixed etiology of liver tumor is also observed, attributing to combined HCC‐CCA [50]. Liver cancer may also derive from precursor and/or embryonic/fetal liver cells in cases of pediatric hepatoblastoma (HBL) [51]. Moreover, several benign liver tumors, for example, hepatocellular adenoma, may undergo malignant transformation [52]. Regardless of the wide diversity of liver tumors, they are often characterized by alterations in key signaling pathways that drive carcinogenesis resulting from mutations of genes involved in chromatin modification (*ARID1A*), protein deubiquitination (*BAP1*), and cell cycle regulation (*CDKN2A*, *CCND1*, *CCNA2*, *KRAS*, *TP53*) [53]. Mutations in *CTNNB1* encoding β‐catenin and *AXIN1* genes are primary genetic alterations in HCC and HBL [51, 54], leading to activation of the canonical WNT/β‐catenin signaling pathway. Although it may take place in the case of CCA as well, CCA is often characterized by different mutations, for example, in *IDH1/2*, *NRAS*, *SMAD4*, and *FGFR2* [55, 56]. Mechanisms of liver carcinogenesis are described in detail in a number of reviews [53, 54, 57, 58], including those focusing on the pathogenesis of liver cancer stem cells [59]. Considering the involvement of lncRNAs in the development of liver cancers, they often affect key cellular processes, for example, activation of WNT/β‐catenin or PI3K/AKT/mTOR pathways, promotion of epithelial–mesenchymal transition (EMT), and triggering receptor tyrosine kinases and cyclin‐dependent kinases (CDK). LncRNAs widely act as important competitive endogenous RNAs (ceRNAs) via sponging different microRNAs and thus switching the expression of target genes, although many of them can directly bind proteins and either block their activity or promote further assembly of protein scaffolds. A variety of lncRNA functions realized in liver carcinogenesis is described in a number of reviews [14, 15, 55, 57, 60, 61].

## Liver cancer biomarkers

Currently, the most widely used diagnostic methods for liver tumors are ultrasound examination and computed tomography scanning combined with alpha‐fetoprotein (AFP) serum analysis [62]. AFP utility is limited due to its low sensitivity and specificity, that might have diagnostic value only in combination with other serum biomarkers, for example, GP73 and DCP [63]. However, DCP is elevated in various liver conditions, whereas GPC3 is expressed not in all HCC cases and can be affected by the tumor microenvironment [58]. Thus, implication of modern novel diagnostic approaches is of great importance, and one of such a direction includes cancer‐associated lncRNAs. Various lncRNAs are reported as potentially useful biomarkers for HCC [5, 16, 64], CCA [19, 65] and HBL [51], and at least some of them might be found in circulating exosomes, thus opening perspective for noninvasive diagnostics [21, 23, 66, 67]. A lot of studies aiming identification and characterization of novel lncRNA transcripts, previously not described in the literature. They typically start from the analysis of RNA‐Seq data obtained for patient tumor samples in comparison to adjacent tissues to find novel up‐ or downregulated lncRNAs. Sometimes the researchers use their own RNA‐Seq data, for example, [68], although most of the works mine transcriptomic data available at The Cancer Genome Atlas (TCGA) [69] or Gene Expression Omnibus database (GEO, www.ncbi.nlm.nih.gov/geo) [70]. Unfortunately, those types of studies mainly focus on lncRNA candidates with highest expression levels and/or significant fold‐change, while typically ignore abundancy of the transcripts and their tissue‐specificity. We cannot find in the literature a single review purposely devoted to liver‐specific lncRNAs, as well as realized that most of known liver‐specific lncRNA candidates were found just by chance.

## Liver‐specific lncRNAs dysregulated in liver cancer

### HULC

To date, *HULC* was shown to be aberrantly expressed in many types of cancers (e.g., breast cancer, pancreatic cancer, bladder cancer, gliomas) and considered a probable diagnostic, prognostic biomarker and therapeutic target for a number of malignancies. Thus, its significance is already much above accounting only liver cancers [24, 25, 26, 27]. However, among all TCGA tumors the highest levels of *HULC* are assigned to HCC (Fig. S3). Its expression in normal tissues is rather low, but it is still more pronounced for liver (Fig. 1). A detailed description of *HULC* functions is beyond the present review, as this lncRNA has rather diverse and multiple functions. It is worth mentioning that it mainly works as a ceRNA, sponging a plenty of microRNAs, suppressing their downstream genes, for example, *FOXM1, VAMP2, HIF‐1α, ATF4, HMGA2, ZEB1*, and affects PKA/CREB, PTEN/AKT/PI3K/mTOR, and E2F1/SPHK1 pathways [27]. *HULC* also upregulates the expression of ubiquitin‐specific peptidase 22 (USP22) resulting in the decrease of ubiquitin‐mediated degradation of SIRT1 [71] and COX‐2 [72]. *HULC* directly binds several proteins (e.g., YBX1 [73], LDHA, PKM2 [74]), and promotes the NF‐kB signaling pathway [75]. *HULC* activation is also essential for viral liver infections [27, 76, 77]. *HULC* significantly contributes to HCC development, progression and chemoresistance, thus being a valuable diagnostic biomarker and therapeutic target for HCC. In the case of CCA, *HULC* expression is remarkably downregulated [78, 79] in line with TCGA data (Fig. S1). Nevertheless, this lncRNA was shown to be a potential prognostic biomarker, as it is strongly associated with poor overall survival of CCA patients [80], which is not the case in terms of HCC (Fig. S2). Altogether *HULC* should be considered more cancer‐specific than liver‐specific lncRNA, with the highest impact to HCC among other types of cancers, but not being a certain characteristic of liver cancer only.

### DBH‐AS1

*DBH‐AS1* was initially considered as lncRNA expressed in hepatitis‐associated liver tumors, although different studies reported different patterns of *DBH‐AS1* dysregulation. Zhang *et al*. found *DBH‐AS1* to be downregulated in 11 HBV‐related HCC samples, while for other 14 HCV‐ and hepatitis delta virus‐related HCC samples dysregulation was inconsistent [81]. In contrast, Bao *et al*. reported up‐regulation of *DBH‐AS1* expression in 21 out of 22 paired HCC/adjacent liver tissues with much higher fold changes for late‐stage tumors [82]. Huang *et al*. analyzed only HCC specimens and observed high levels of *DBH‐AS1* positively associated with increased tumor size and HBV infection. *DBH‐AS1* was significantly upregulated *in vitro* upon expressing HBV viral protein HBx by lentivirus infection, and its high expression levels in HCC specimens were associated with high *HBx* mRNA levels [83]. Two independent studies reported high expression levels of *DBH‐AS1* in different cancer hepatocyte cell lines [82, 83], including HСС‐derived Huh‐7, Hep3B and MHCC97‐H, and HBL‐derived Hep‐G2 (previously considered as originated from HCC, but now annotated as HBL [84]). Huang *et al*. [83] obtained *DBH‐AS1* knockdown in both in Hep3B and Hep‐G2, whereas *DBH‐AS1* overexpression was investigated in HBL‐derived Hep‐G2. ShRNA‐mediated knockdown of *DBH‐AS1* in Hep3B led to decreased viability and increased apoptotic rates of cells. Overexpression of *DBH‐AS1* in Hep‐G2 enhanced their proliferation and colony formation ability, as well as in increased tumor growth in nude mice. High levels of *DBH‐AS1* also elevated the expression of oncogenic cell cycle regulators CDK6, CCND1, CCNE1, repressed cyclin‐dependent protein kinase inhibitors p16, p21, p27 and activated MAPK signaling pathways manifesting as up‐regulation of p‐ERK, p‐p38 and p‐JNK [83]. However, data for *DBH‐AS1* overexpression were obtained in HBL‐derived Hep‐G2 cell line only. Bao *et al*. performed *in vitro* experiments for knockdown and overexpression of *DBH‐AS1* in HCC cell models Huh‐7 and Hep3B and revealed this lncRNA binding miR‐138 [82]. Expression levels of miR‐138 and *DBH‐AS1* were also negatively associated in HCC patient samples. Ectopic miR‐138 expression significantly reduced cell viability and induced apoptosis in Huh‐7 and Hep3B cells, but *DBH‐AS1* co‐expression effectively attenuated miR‐138‐elicited effects, including restoration of phosphorylation levels of proteins of FAK/Src/ERK pathway affected by miR‐138 both *in vitro* and *in vivo* [82]. Up to date miR‐138 is known to participate in a number of key molecular mechanisms essential for HCC, for example, suppression of HIF‐1α/VEGFA pathway. It is also widely considered as potential therapeutic target [85], enhancing *DBH‐AS1* perspective application. However, it came to our attention, that *DBH‐AS1* is expressed in different normal human tissues, although its highest levels are indeed attributed to liver (Fig. 1). TCGA datasets also demonstrate moderate *DBH‐AS1* expression levels across various tumors (Fig. S3). In HCC TCGA dataset *DBH‐AS1* seems not to be affected (Fig. S1), thus diagnostic value of *DBH‐AS1* seems to be disputable. Recent studies also reported up‐regulation of *DBH‐AS1* in osteosarcoma [86] and diffuse large B‐cell lymphoma [87]. Altogether these data do not support sound liver‐ or HCC‐specificity of *DBH‐AS1*.

### LINC01554

*LINC01554* was found by RNA‐Seq analysis of paired HCС/normal liver patient samples and further detected in a set of hepatocyte cell lines (HСС‐derived Huh‐7, Hep3B and HBL‐derived Hep‐G2) [88]. Highest expression of *LINC01554* was observed in the immortalized normal liver cell line (MIHA) consistent with significant down‐regulation of this lncRNA in cancerous hepatocyte cell lines and HCC samples. A single 1943 nt transcript was identified by 5′/3′‐RACE and confirmed by northern blotting. In line with cytoplasmic localization of *LINC01554* the authors identified miR‐365a‐3p that downregulated its expression. Negative association between *LINC01554* and miR‐365a‐3p expression was found in HCC tumor samples as well [88]. There were also a number of functional experiments performed by the authors [88], including identification of its protein partner PKM2, however, all HCC cell lines used in that study were recently reported to be actually HeLa‐derived [89]. Nevertheless, inhibited proliferation of cell lines overexpressing *LINC01554* was shown in another studies in the models of conventional HCC cell lines Hep3B and Huh‐7 [90], HCCLM9 [91] and HCCLM3 [92]. Hao *et al*. identified that *LINC01554* sponges miR‐3681‐3p and prevents its binding to *NGFR* mRNA interfering negative regulation of *NGFR* expression. Downstream activation of *NGFR* suppressed cell proliferation and invasiveness [89]. Li *et al*. found that *LINC01554* overexpression promotes G0/G1 arrest, but does not affect apoptosis. By a number of western blots, it was showed that *LINC01554* overexpression increased amounts of TJP1 (ZO‐1) and E‐cadherin, while decreased N‐cadherin and vimentin expression levels, as well as inhibited expression of AKT, p‐AKT, β‐catenin, and p‐GSK3β [91]. Recently Ren *et al*. demonstrated *LINC01554* to sponge miR‐148b‐3p preventing mediated inhibition of *EIF4E3* expression [93]. In contrast to previous study, *LINC01554* overexpression was found to promote cell apoptosis. The authors confirmed significant up‐regulation in expression levels of key apoptosis‐related genes such as BAX, BCL2/BAX, p53 and cleaved‐Caspase3 [93]. Taking together these data more appeal to tumor‐suppressive role of *LINC01554*, which is likely gained by inhibition of WNT and PI3K/AKT signaling pathways and suppression of EMT.

Although exact mechanisms of *LINC01554* functioning need further investigation, its' high prognostic value in terms of HCC is reproducibly revealed in every study. Low expression of *LINC01554* in HCC tissues is strongly associated with poorer survival of patients (Fig. S2), that was confirmed in a number of independent studies [88, 91, 92]. Downregulation of *LINC01554* is associated with adjacent organs invasion, tumor size, staging and higher risks of tumor recurrence [88, 91]. *LINC01554* was also identified by single‐cell sequencing of HCC samples among 10 top survival‐related hub genes for diagnosis and prognosis of HCC [94]. *LINC01554* was considered in all abovementioned studies as liver‐specific [91] or at least liver‐enriched [92] lncRNA. GTEx data shows very low expression of *LINC01554* in other tissues, along with its highest abundancy in healthy liver tissues (Fig. 1). We suggest low expression levels of *LINC01554* observed in different TCGA tumor datasets (Fig. S3) also reflect some general background *LINC01554* amounts, as this lncRNA is downregulated in HCC and CCA (Fig. S1). Further investigations are necessary to validate, whether *LINC01554* expression in other tissues is functional, or it results from background transcriptional activity within this locus. Notably, *LINC01554* expression levels in healthy liver is higher in 1–2 orders in comparison to other tissues (Fig. 1). Thus, we suggest it should be considered as liver‐specific lncRNA.

### LINC01018

*LINC01018* was found as downregulated lncRNA in HBV‐associated liver cancer samples. It was assigned as a liver‐specific transcript co‐expressed with some cancer development genes [95]. Constructions of competitive endogenous RNA (ceRNA) networks predicted *LINC01018* to sponge miRNA‐574‐5p and to affect glucose‐6‐phosphatase catalytic subunit (G6PC) [95]. However, further *in vitro* studies performed on HCC cell line Hep3B revealed *LINC01018* sponging another one target, miR‐182‐5p, highly expressed in HCC [96]. Overexpression of *LINC01018* resulted in competitive binding of miR‐182‐5p and downstream activation of *FOXO1* expression, that also led to reduction of cancer cell proliferation and increased apoptosis. Western blots confirmed activation of BAX and decrease of BCL‐2 upon *LINC01018* overexpression. While all effects were reversible upon up‐regulation of miR‐182‐5p. Finally, experiments on xenograft mice overexpressing *LINC01018* showed up‐regulation of *FOXO1* expression and significant decrease of tumor size. Down‐regulation of *LINC01018* was also demonstrated in 72 pairs of HCC patient samples [96]. Diagnostic value of this lncRNA was also confirmed in terms of HBV‐related HCC [97]. Perspective application of *LINC01018* as a prognostic biomarker was claimed in [98], in line with prediction of hsa‐miR‐197‐3p/GNA14 ceRNA network. Although highest expression levels of *LINC01018* are assigned to the liver, some pronounced expression is also observed in ovaries, pancreas, and brain tissues (Fig. 1). Several studies reported oncosuppressor role of *LINC01018* realized in gliomas by targeting miR‐942‐5p and thus activating downstream *KNG1* expression, as well as abovementioned miR‐182‐5p [99, 100]. Prognostic value of *LINC01018* downregulation and its therapeutic potential to inhibit miR‐182‐5p was also considered in terms of prostate cancer [101], where this lncRNA also shows high expression similar to that in HCC (Fig. 2). Considering these facts *LINC01018* seems not to be specific for liver and/or liver cancers. Although we should highlight its potential prognostic value, as its high expression levels are strongly associated with better overall survival of HCC patients (Fig. S2), [98].

### LINC01348

There is only a single study characterizing this lncRNA [102]; however, the authors performed a comprehensive investigation, revealed exact functions of *LINC01348* and showed its oncosuppressive role in HCC. *LINC01348* is a single 1000 nt transcript comprising of four exons (NR_027454). RACE assays performed in HBL‐derived Hep‐G2 and HCC‐derived Huh‐7 cell lines uncovered the existence of two additional minor transcripts with truncated 5′‐end and 40 nt internal deletion, but also fully confirmed the abundant full‐length isoform [102]. Remarkable down‐regulation of *LINC01348* was observed in HCC tissues by RT‐qPCR and additionally confirmed by RNA‐immunohistochemistry. Notably, *LINC01348* levels in adjacent liver samples were high enough to be clearly visualized in nuclei. High expression of *LINC01348* was associated with better overall survival of patients with HCC and negatively correlated with pathological stage, grade of tumor and AFP serum levels. Stable *LINC01348* knockdown in HCC cell lines Hep3B and J7 and overexpression in HCC‐derived Huh‐7 and HBL‐derived Hep‐G2 revealed oncosuppressive function of this lncRNA regardless its exact isoform. Elevated levels of *LINC01348* promoted suppression of cell growth, migration, and invasion [102]. Finally, the splicing factor 3B subunit 3 (SF3B3) was found by RNA pull‐down and by RNA immunoprecipitation (RIP) assays as a *LINC01348* main protein partner in HCC cell model J7 [102]. The authors specified exact *LINC01348* region (186–476 nt) responsible for direct interaction with SF3B3 and blockage of its further binding to SF3B1. SF3B3‐SF3B1 splicing complex promotes the inclusion of *EZH2*‐mRNA 14th exon—a protumorigenic event, which significantly contributes to cell proliferation and metastasis [103]. *LINC01348*‐mediated inhibition of the *EZH2* pre‐mRNA splicing affects downstream JNK/c‐JUN/SNAI1 pathway resulting in reduction of cell metastasis. The authors also uncovered a link of *LINC01348* with HBV‐mediated HCC. Overexpression of C‐terminal domain truncated HBx suppressed *LINC01348* expression by enhancing c‐JUN binding to its promoter fragment, that additionally contributes to activation of oncogenic pathways to promote tumor formation and HCC development [102]. Considering pronounced expression of *LINC01348* predominantly in liver tissues (Fig. 1) and its involvement in such essential processes of cancer suppression, it might possess perspective utility for development of therapeutic strategies for HCC. Notably, *LINC01348* is significantly downregulated in liver cancers, but not strongly affected in other liver diseases, such as viral infection, cirrhosis, NAFLD, etc., (Fig. 2).

### LINC02362

*LINC02362* is a liver‐specific lncRNA reported in two independent studies [104, 105]. It was shown to be significantly downregulated in HCC tissues, and its high levels were associated with a better patient prognosis [104], consistent with TCGA data (Fig. S2). Lower *LINC02362* expression was observed in TNM stage II and III of HCC tumors, and also in patients with microvascular or macrovascular invasion and those with elevated AFP serum levels. Ectopic overexpression of *LINC02362* in HCC cell lines Hep3B and PLC/PRF/5 resulted into cell cycle arrest, inhibited proliferation, migration, and invasiveness. It also affects EMT due to upregulation of E‐cadherin and downregulation of N‐cadherin and vimentin [104]. *LINC02362* was shown to sponge and suppress miR‐516b‐5p—the oncogenic miRNA related to poor prognosis of HCC patients. Moreover, *LINC02362* could be also suppressed by the overexpression of miR‐516b‐5p, so these two molecules regulate each other. miR‐516b‐5p binds 5’‐UTR of *SOCS2* (suppressor of cytokine signaling 2) mRNA promoting its degradation. Both mRNA and protein levels of SOCS2 were upregulated upon *LINC02362* overexpression, in line with positive correlation between their expression levels observed in HCC patients [104]. Thus, *LINC02362* by sponging miR‐516b‐5p protects SOCS2, which is a well‐known tumor suppressor inhibiting HCC cell proliferation [106]. *LINC02362* was also revealed to sponge miR‐18a‐5p and promote expression of its downstream target FDX1—a key regulator in cuproptosis and upstream regulator of protein lipoylation, which expression levels are associated with improved survival of HCC patients. *LINC02362* knockdown in HCC cell lines MHCC97‐H and Huh‐7 significantly reduced copper concentration in elesclomol‐Cu treated HCC cell lines and increased their chemoresistance to oxaliplatin [105], thus presenting a novel pathway for potential therapeutic application of *LINC02362*. According to GTEx data *LINC02362* highest expression levels are attributed to liver tissues, whereas some amounts are also detected in colon, small intestine, and EBV‐transformed lymphocytes (Fig. 1)—further research is needed to clarify whether *LINC02362* expression is functional outside liver. *LINC02362* in significantly downregulated in liver cancers (Fig. S1), but highly expressed in non‐malignant liver diseases and conditions, with the exception of ALD and fetal liver (Fig. 2).

### LINC01146

*LINC01146* was identified as a liver‐specific lncRNA with probable involvement in the occurrence and development of HCC [107]. According to the TCGA, *LINC01146* is significantly decreased in HCC compared to normal liver tissues (Fig. S1) and low *LINC01146* expression is associated with poor patient survival (Fig. S2). These data were validated on an independent collection of HCC patient samples, and the expression of *LINC01146* was found to be negatively associated with tumor size, grade, microvascular invasion, number of satellite nodules, and HBV infection [107]. High expression of *LINC01146* in HCC tumors was associated with much longer overall survival in patients. The expression of *LINC01146* was also verified in a number of HCC cancer cell lines (Huh‐7, Hep3B, HCCLM3, MHCC97‐H, SNU‐423, SNU‐449) and further overexpression experiments in Huh‐7 and MHCC97‐H demonstrated inhibited cell growth and proliferation. Reversed effects were observed in the case of *LINC01146* knockdown in Huh‐7 and Hep3B. *In vivo* experiments in mice confirmed *LINC01146* function as a suppressor of tumor growth. The search for co‐expressed genes revealed a number of targets related to *LINC01146*, and the strongest correlations were found for genes *FETUB* and *TTR*, which encode highly liver‐specific proteins fetuin B and transthyretin [107].

Although *LINC01146* has highest expression levels in normal liver tissues, it is also specified for moderate expression in pancreas islets, testis and prostate (Fig. 1), whereas among different TCGA datasets (Fig. S3) *LINC01146* is a main characteristic both for HCC and prostate cancer (PC). Indeed, recent study reported *LINC01146* involvement in carcinogenesis upon PC [108]. In contrast to liver cancers in PC specimens, *LINC01146* was shown to be upregulated. It was revealed that *LINC01146* acts as an oncogene and facilitates viability, proliferation, migration and invasion in PC cells *in vitro*, as well as promotes tumor growth *in vivo* [108]. *LINC01146* was shown to be located in cytoplasm and interacts with F11 receptor (F11R), a type I transmembrane protein, that is positively linked to malignant progression and poor prognosis of PC. *LINC01106* activates F11R, and they both are regulated by TGF‐β1. Besides, overexpression of *LINC01106* downregulated E‐cadherin, BAX, and Cleaved caspase‐3, while upregulated N‐cadherin, vimentin, and PCNA [108]. These data confirm the oncogenic role of *LINC01106* in PC. Thus, *LINC01106* represents an illustrative example of lncRNA that has diametrically opposite functions realized in different human organs and tumors. Altogether *LINC01146* should not be considered as a liver‐specific lncRNA.

### lncRNA‐DAW

We also found one more interesting example of the claimed liver‐specific lncRNA, which seems to be expressed not only in liver. There is only a single study investigating this lncRNA—in 2021 Liang *et al*. discovered a novel liver‐specific super‐enhancer in the locus 20q13.12 [109]. Further analysis revealed lncRNA *LINC01430* (renamed to *lnc‐DAW*) and assigned to a single transcript of 442 nt (NR_109893.1) localized in the nucleus. Previous annotation reported high liver‐specificity of NR_109893.1. However, we found out this gene to be re‐annotated now within ENSG00000168746 (current name *LINC01620*). Thus, according to modern annotation *lnc‐DAW* is a single transcript ENST00000415299.2 among five isoforms of *LINC01620*. Previous *LINC01430* annotation (ENSG00000237907) is now discarded from most of genomic databases. We explored individual expression of each transcript in GTEx database and in FLIBase (www.flibase.org, [110]). All *LINC01620* transcripts show high expression levels in testis, and only a single one isoform is expressed both in testis and liver (Fig. S4). Thus we suggest overall expression levels reported for *LINC01620* by GTEx being rather representative (Fig. 1). There are yet no experimental confirmation for expression of *LINC01620* in testis, whereas high amounts of *lnc‐DAW* were found by RT‐qPCR in HCC cell lines Hep3B and Huh‐7 [109]. Liang *et al*. also shown that *lnc‐DAW* overexpression in HCC‐derived PLC/PRF/5 and HBL‐derived Hep‐G2 promoted cell proliferation *in vitro*, as well as *lnc‐DAW* overexpression in HBL‐derived Hep‐G2 potentiated *in vivo* tumor growth and metastasis. RNA‐Seq analysis of both overexpressing cell lines (Hep‐G2 and PLC/PRF/5) revealed association of *lnc‐DAW* levels with WNT2, and thus, it was named *lnc‐DAW* aka “Distant Activator of WNT2”. The authors made further comprehensive research and showed direct interaction of *lncRNA‐DAW* with EZH2 resulting into its CDK1‐mediated phosphorylation, ubiquitination, and degradation, that releases inhibition of WNT2 transcription and activates WNT/β‐catenin pathway. Up‐regulation of *lncRNA‐DAW* expression was found in 50 paired (tumor/adjacent) HCC cases, which was also associated with elevated WNT2 expression levels in tumor samples. Therefore, this lncRNA was proposed as a novel oncogenic target and putative clinical diagnosis biomarker for HCC [109]. However, we noted that *lncRNA‐DAW/LINC01620* in general has the lowest expression levels among all lncRNAs described in the present review, regardless the type of liver tumor or disease (Fig. 2, Fig. S3). It seems to be downregulated both in HCC and CCA in TCGA datasets, even if we take into consideration only single *lncRNA‐DAW* transcript (*LINC01430*, Fig. S1), that is contradictory to the data reported in [109]. Further investigations are needed to clarify these issues. However, it seems like *lncRNA‐DAW* cannot be considered as liver‐specific lncRNA.

### FAM99A

*FAM99A* is a novel lncRNA actively investigated in terms of HCC. Although showing some moderate expression levels in spleen and testis, its highest expression attributed to normal liver (Fig. 1), in line with significant down‐regulation in HCC and CCA (Fig. S1) but not in other liver diseases and conditions (Fig. 2). Low *FAM99A* expression is significantly associated with vascular invasion, advanced histological grade of HCC tumors and worse patient survival [110, 111, 112]. *FAM99A* overexpression remarkably inhibited viability, migration, and invasion abilities of HCC cell models Hep3B and Huh‐7 [110, 111], while *FAM99A* knockdown facilitated the viability and clonogenity of these cell lines [111]. In HCC xenograft mice models *FAM99A* overexpression dramatically inhibited tumor growth, decreased cell necrosis, and infiltration of tumor tissues [111]. *FAM99A* was found to localize in nuclei, and RNA FISH (fluorescence *in situ* hybridization) and further RNA pull‐down experiments led to identification of seven enriched proteins: poly (rC) binding protein 1 (PCBP1), splicing factors SRSF5 and SRSF6, YBX1, IGF2BP2, HNRNPK and HNRNPL [111]. It was proposed, that *FAM99A* target miR‐92a, a known regulator of E‐cadherin. Unfortunately, corresponding *in vitro* experiments [110] were performed in misclassified SMMC‐7721 and SK‐HEP‐1 cell lines [89], therefore they cannot be considered in terms of *FAM99A* function in hepatocytes or HCC. *FAM99A* was also found to be upregulated upon treatment of cancerous hepatocytes (HBL‐derived Hep‐G2 and HCC‐derived HCCLM3) by icaritin—a natural chemotherapeutical agent isolated from *Epimedium* plants which was under clinical trials for HCC therapy [113]. Icaritin reduces viability and proliferation of both cell lines by repressing JAK2/STAT3 pathway though reduction of GLUT1, and thus inhibits Warburg effect. *In vitro* experiments in Hep‐G2 and HCCLM3 cell lines revealed that this function is mediated by *FAM99A* upregulation via its interacting with miR‐299‐5p and promoting SOCS3 expression, as well as by inhibiting STAT3 phosphorylation, that altogether represses JAK2/STAT3 pathway. These findings were confirmed in xenograft mice models, which showed upon *FAM99A* overexpression (performed by HBL‐derived Hep‐G2 cell model) reduced tumor growth, downregulation of GLUT1 and blocked nuclear translocation of STAT3 [113].

### FAM99B

*FAM99B* is a single 1066 nt transcript highly abundant in hepatic tissues, although some background expression was also evident in spleen and testis (Fig. 1). *FAM99B* is prominently decreased in HCC according to TCGA data (Fig. S1), that was also shown by the comprehensive analysis of available GEO datasets and validation by RT‐qPCR of paired HCC/adjacent liver samples [114, 115, 116]. Low *FAM99B* expression was associated with vascular invasion, advanced histologic grade and tumor stage and worse patient survival [114, 115, 116]. Stable overexpression of *FAM99B* in HCC cell line HCCLM3 remarkably inhibit cell proliferation rates, migration, and invasion [115]. Similar effect was observed in the independent study by *FAM99B* overexpression in HСС‐derived Huh‐7 and HBL‐derived Hep‐G2 cell lines [116]. Oncosuppressive role of *FAM99B* was also confirmed in mice [116]. Previously function of *FAM99B* was studied by obtaining *FAM99B*‐containing exosomes (Exo‐*FAM99B*) derived from human umbilical cord mesenchymal stem cells (hUC‐MSCs). Exo‐*FAM99B* successfully entered HCC cells MHCC97‐H and induced cell cycle arrest at the G0/G1 stage, promoted apoptosis and suppressed migration and invasiveness [115]. Recently, He *et al*. [116] discovered that *FAM99B* inhibits ribosome biogenesis in cancer cells due to mediated cleavage of dead‐box Helicase 21 (DDX21). Notably, most of the *in vitro* experiments were performed in parallel in Huh‐7 and Hep‐G2 cell lines, thus being eligible for both HCC and HBL cancer models. *FAM99B* is localized in nuclei together with DDX21 and RNA pull‐down and RIP assays revealed *FAM99B* direct binding to the C‐terminal domain of DDX21. *FAM99B* recruits XPO1 exportin, which translocates DDX21 to the cytoplasm, where DDX21 is degraded via caspase‐3/caspase‐6‐mediated cleavage. Comparative transcriptomic analysis of genes, differentially expressed upon *FAM99B* overexpression *vs* DDX21 knockdown in HCC cell line Huh‐7, revealed strongly affected translation machinery and inhibition of global protein expression levels. It was shown, that *FAM99B* overexpression led to accumulation of 47/45S and 30S pre‐rRNAs, but to decrease of 21S and 18S‐E pre‐rRNAs. Thus, FAM99B and DDX21 can regulate the processing of pre‐rRNAs at the A site. ChIP‐Seq (chromatin immunoprecipitation with further sequencing) analysis showed DDX21 abundancy at the promoters of genes encoding ribosomal proteins RPS29 and RPL38, and DDX21 knockdown resulted in significant downregulation of the mRNA and protein expression of RPS29/RPL38. Altogether, FAM99B affects ribosome biogenesis by regulating rRNA processing and RPS29/RPL38 transcription via DDX21. By the analysis of *FAM99B* deletion mutants, the authors [116] determined its exact functional domain, comprising of short region from 65 to 146 nt (*FAM99B* ^*65–146*^), and its overexpression perfectly restored full‐length *FAM99B* phenotype. Finally, *FAM99B* ^*65–146*^ was shown to have strong therapeutic effect on orthotopic xenograft mice models of HCC (Huh‐7‐derived), which were treated by weekly injections of 5 mg·kg^−1^ doses of *FAM99B* ^*65–146*^ molecule, conjugated with GalNAc for target delivery in liver (see Introduction). Treated group of mice showed lower tumor growth rates, decreased KI67 and DDX21 expression in tumors and fewer intrahepatic metastases, along with desirable accumulation of GalNAc*‐FAM99B* ^*65–146*^ in liver tissues, but not in lungs, as well as the lack of toxicity [116]. To our knowledge, this is the first one example of liver‐specific lncRNA, which showed such a high potential in terms of probable future applications for liver cancer treatment.

### LINC01093

In 2019, He *at el*. reported a novel liver‐specific lncRNA *LINC01093* remarkably downregulated in HCC and in alcoholic hepatitis and HBV‐associated acute liver failure samples [117]. Expression levels of *LINC01093* were negatively associated with TNM stage of HCC patients, existence of cancer embolus, overall survival and rates of time to recurrence [117]. Another study demonstrated downregulation of *LINC01093* expression in HBL, non‐malignant hepatocellular adenoma and focal nodular hyperplasia in comparison to adjacent liver tissues, whereas CCA samples were characterized by total lack of *LINC01093* [78]. *In vitro* experiments (shRNA‐mediated knockdown *vs* overexpression) showed oncosuppressor functions of *LINC01093* affecting proliferation of HCC cell line Huh‐7 [117]. These results were also confirmed *in vivo* on xenograft mice models, and those tumors overexpressing *LINC01093* were dramatically smaller and decreased numbers of lung metastatic nodules were observed. *LINC01093* was mostly found in cytoplasm and RNA pull‐down and RIP assays revealed its protein partner insulin‐like growth factor 2 mRNA‐binding protein 1 (IGF2BP1)—highly conserved oncofetal protein. *LINC01093* prevents IGF2BP1 binding to its downstream target *GLI1* mRNA and promotes its degradation. GLI1 is a well‐known oncogenic transcriptional activator and its *LINC01093*‐mediated inhibition led to down‐regulation of such key proteins as EGFR, FOXM1, VEGF and BCL‐XL [117]. *LINC01093* functions were also investigated in mice models for liver diseases. *LINC01093* overexpression in alcoholic hepatitis mice models effectively suppressed apoptosis and promoted proliferation by inhibiting the ICAM‐1‐mediated NF‐κB signaling pathway [118]. In contrast, downregulation of *LINC01093* in mice models of liver fibrosis promoted hepatocyte apoptosis mediated by increasing the degradation and ubiquitination of SIRT1. Direct interaction of SIRT1 and *LINC01093* was confirmed by RNA pull‐down and RIP experiments [119]. Recently, *LINC01093* was identified to be strongly associated with HBV‐liver cancer prognosis as upregulation of this lncRNA presented higher overall survival rates [120]. *LINC01093* overexpression independently reduced the number of copies of HBV DNA infecting HBL‐derived Hep‐G2/2.2.15 cell line, and ELISA experiments showed decreased levels of viral factors HBsAg and HBeAg [120]. Thus, *LINC01093* might be considered as potential therapeutic target for HBV‐associated liver cancer. According to GTEx data *LINC01093* shows pronounced specificity to liver tissues (Fig. 1). Its application as liver cancer biomarker is discussible, as decreased *LINC01093* expression is shown also in ALD (Fig. 2), however among other types of tumors *LINC01093* seems to be detected mainly in HCC (Fig. S3).

### LINC02428

There is only a single study characterizing this lncRNA, where its expression was experimentally confirmed by RT‐qPCR in eight human normal clinical liver samples and in 13 paired (tumor/adjacent) HCC samples [121]. Strong down‐regulation of this lncRNA was shown in HCC cancer tissues [105], in line with the TCGA dataset (Fig. S1). Patients with high expression of *LINC02428* had better prognosis and overall survival [121]. Expression of *LINC02428* in most of screened hepatocyte cell lines was nearly not detectable, and only MHCC97‐H, MHCC97‐L, and Huh‐7 were somehow *LINC02428*‐positive, but the highest detected amount of this lncRNA was still significantly less compared to liver samples. *LINC024283* overexpression in HCC cell lines Huh‐7 and Hep3B suppressed cell proliferation *in vitro* and inhibited tumor growth in mice. *LINC02428* overexpression also resulted in reduced lung metastasis, decreased expression of the proliferation markers KI67, PCNA, N‐cadherin, and vimentin in tumor tissues. RNA FISH assays revealed cytoplasmic localization of *LINC02428* and further experiments identified its partner IGF2BP1, that binds 801–1348 nt region of this lncRNA. Interestingly, *LINC02428* overexpression affects both protein and mRNA levels of *IGF2BP1*. Interaction between *LINC02428* and IGF2BP1 interferes its downstream binding to *KDM5B* mRNA and attenuates its stabilization. As far as KDM5B is a transcription factor activating IGF2BP1 promoter, this regulation exists as a positive feedback loop, and *LINC02428* blocks it, thus inhibits HCC occurrence and development [121]. Together with 5 other novel lncRNAs *LINC02428* was used for construction of HCC prognostic signature [122]. According to GTEx data *LINC02428* indeed is highly liver‐specific (Fig. 1).

### LIVAR

*LIVAR* is a lncRNA mentioned in a single publication only. It was discovered in 2019 by Atanasovska *et al*. by microarray‐based gene expression profiling of liver biopsies samples obtained from NASH patients [123]. The authors found a liver‐specific lncRNA *RP11‐484N16* (current name *LIVAR*), which expression was associated with NASH grade, lobular inflammation, NAFLD activity score and fibrosis. Full transcript (633 nt, 2 exons) was confirmed by Sanger sequencing. It was actively expressed in all tested hepatocyte cell lines (HCC‐derived Hep3B, HBL‐derived Hep‐G2, and immortalized human hepatocytes IHH‐A5 [124]) and primary human hepatocytes, with highest levels observed for Hep‐G2. Fractionation experiments showed cytoplasmic localization of *LIVAR*. Surprisingly, shRNA‐mediated knockdown of *LIVAR* resulted in lethal phenotype of Hep3B and Huh‐7 HCC cell lines, as well as into reduced growth of Hep‐G2 and IHH. Based on these data, this lncRNA was renamed for *LIVAR* aka “LIver cell Viability Associated lncRNA”. Further RNA‐Seq analysis of *LIVAR* knockdowns in Huh‐7 and Hep‐G2 revealed a lot of downregulated genes involved in cell death pathways, anti‐apoptotic processes, and translation elongation. Most of upregulated genes were enriched for the oxidation–reduction pathways. Significant reduction of intact poly(ADP‐ribose) polymerase 1 PARP1, a key enzyme in DNA repair, and increase of necrotic nuclei were also observed as hallmark features of apoptosis upon knockdown experiments in Huh‐7 and IHH [123]; however, exact *LIVAR* function remains elusive so far. According to TCGA datasets *LIVAR* expression is downregulated upon CCA and nearly no affected upon HCC development in comparison to adjacent tissues (Fig. S1). We also noted high expression levels of *LIVAR* in HBL tissues according to the data from GepLiver database (Fig. 2), which is in line with its highest amounts found *in vitro* in Hep‐G2 [123].

### CPS1‐IT1

Carbamoyl‐phosphate synthase 1 (CPS1) is a mitochondrial enzyme participating in the urea cycle with predominant expression in liver. Deficiency of CPS1 may be a crucial error in hepatocyte metabolism found in newborns with urea cycle disorders. It leads to acute hyperammonemia, that causes various dysfunctions of central neural system [125]. *CPS1* intronic transcript 1(*CPS1‐IT1*) is encoded within one of big *CPS1* introns as a 2306 nt single unspliced isoform. Expression of *CPS1‐IT1* is also exclusive in liver tissues (Fig. 1). According to TCGA datasets *CPS1‐IT1* is downregulated both in HCC and CCA (Fig. S1), whereas its expression is not detected among other tumors (Fig. S3). *CPS1‐IT1* was essentially decreased in HCC patient samples compared to normal liver tissues, and low *CPS1‐IT1* expression levels were associated with poor patient survival [126]. Overexpression of *CPS1‐IT1* in HCC‐derived cell line J7 significantly reduced cell proliferation as it repressed expression of EMT‐promoting proteins N‐cadherin, vimentin, SNAIL, and TWIST and promoted E‐cadherin and occludin expression. Wang *et al*. also performed RNA pull‐down and RIP experiments unraveled the association of *CPS1‐IT1* with protein chaperone HSP90 preventing its binding to HIF‐1α, and also tumor suppressor role of *CPS1‐IT1* was also validated *in vivo* on xenograft mice [126]. However, these particular data were obtained in previously misclassified cell line SK‐HEP‐1 [89] and cannot be considered in terms HCC and/or hepatocytes. Further studies revealed *CPS1‐IT1* activation upon melatonin treatment in HCC‐derived Huh‐7 and HBL‐derived Hep‐G2 cells [127]. Melatonin significantly inhibited the proliferation, migration, and invasion of cancer cells and suppressed EMT. Such an effect was modulated by decrease of HIF‐1α expression, that was a downstream consequence of *CPS1‐IT1* activation. Analysis of *CPS1‐IT1* promoter regions revealed several binding sites for transcription factor FOXA2, and further melatonin‐induced activation of FOXA2 was determined. *In vivo* xenograft mice HCC model (originated from Huh‐7 overexpressing *CPS1‐IT1*) upon melatonin treatment demonstrated inhibited tumor growth, promoted cell differentiation and downregulated expression of EMT‐promoting proteins as well as upregulated E‐cadherin levels [127]. Altogether, *CPS1‐IT1* demonstrates strong oncosuppressive function in HCC and high perspective prognostic potential along with probable therapeutic value.

### Other lncRNAs

We also found a number of publications which only mentioned some liver‐specific lncRNAs with no exploration of their properties and functions. For example, in the article devoted to *LIVAR*, two additional novel lncRNAs, *MAPKAPK5‐AS1* and *RP4‐763G1.2*, associated with NASH and NAFLD were reported [123]. *MAPKAPK5‐AS1* (ENSG00000234608) was shown to promote HCC progression through the LAGL2/HIF‐1α signaling loop [128]; however, it has no tissue‐ or cancer‐specificity. In contrast, *RP4‐763G1.2*, also known as *LINC01702*, indeed has pronounced expression in liver, although some moderate expression levels in kidney (Fig. 1), further investigations are needed to verify its expression in liver cancers.

Tan *et al*. reported two novel liver‐specific lncRNAs *NONHSAT059247.2* and *NONHSAT013897.2* downregulated in HCC, which demonstrated perspective diagnostic performance [129]. *NONHSAT059247.2* is a *SMUG1P1* pseudogene (ENSG00000267444), that indeed has exclusive liver‐specificity (Fig. 1). *NONHSAT013897.2* appeared to be a novel gene ENSG00000301241, not yet described in GTEx and TCGA databases, besides NONCODE database (www.noncode.org, [130]) reports expression of *NONHSAT013897.2* in human breast only.

*LINC02499* was found by comprehensive RNA‐Seq analysis of human tissues that is restrictedly expressed in the liver and kidneys only [131], with much higher expression in liver tissues (Fig. 1). *LINC02499* expression was found to be remarkably lower in HCC compared to noncancerous tissues (Fig. S1), which was confirmed by RT‐qPCR in 80 paired tumor/normal HCC patient samples [131]. Low level of *LINC02499* was associated with high AFP level, microvascular invasion, the number of satellite nodules, advanced tumor grade, and poor patient survival. Overexpression of *LINC02499* significantly inhibited the proliferation of HCC‐derived HCCLM3 and SNU‐449 cell lines, suppressed cell migration and invasion, and opposite effects were observed upon *LINC02499* knockdown [131]. Co‐expression networks showed a significant association of *LINC02499* with the expression of CYP3A5 in the liver [132]. Pathway enrichment analyses revealed potential target genes of *LINC02499* as involved in complement and coagulation cascades and butanoate metabolism [131]. However, these predictions need further experimental validation. Nevertheless, *LINC02499* was successfully used together with other lncRNAs for the construction of prognostic signatures for HCC [133, 134] and considered a prospective biomarker. Its high expression indeed is strongly associated with better survival of HCC patients (Fig. S2).

*RP11‐116D2.1* was discovered by expression profiling analysis of 14 pairs of HCC/adjacent samples as a significantly downregulated transcript, that was also confirmed by RT‐qPCR [135]. Further study “re‐opened” *AC115619.1* (alias name for *RP11‐116D2.1*) among other lncRNAs differentially expressed in HCC, and showed its expression to be negatively associated with tumor grade and invasion [122]. *AC115619.1* overexpression inhibited the proliferation of HCC‐derived SNU‐449 and HBL‐derived Hep‐G2 cells [122]. KEGG pathway enrichment predicted *AC115619.1*‐related genes involved in spliceosome assembly [122, 135] and endocytosis and cell cycle [122], however, there is no any experimental evidence for these functions. Nevertheless, *AC115619.1* has an exclusive liver‐specific expression in normal human tissues (Fig. 1) and was shown to have positive prognostic value for HCC (Fig. S2), indicating its role as probable oncosuppressor [122, 135].

LncRNAs *HELIS* (aka “healthy liver specific”) was reported as a highly liver‐specific lncRNA, that is confirmed by GTEx data (Fig. 1). Its expression levels were investigated in 82 paired (tumor/adjacent) patient samples representing different liver lesions: HCC, CCA, combined HCC‐CCA, HBL, and benign tumors [78]. *HELIS* was not expressed in non‐hepatocyte tumors (HBL, CCA), in contrast to benign lesions, which were characterized by only weak decrease of *HELIS* expression. *HELIS* expression was also correlated with differentiation grade of HCC in a manner that poorly differentiated tumors lacked *HELIS*, whereas highly‐differentiated HCC tumors still expressed low amounts of *HELIS* [78].

*AC008549.1* (*CTC‐505O3.2*) was found in children's fibrosis known as biliary atresia [136]. It was significantly downregulated in choledochal cyst liver tissues. According to the TCGA datasets *AC008549.1* is expressed only in HCC (Fig. S3) and is significantly downregulated both in HCC and CCA (Fig. S1), besides its expression is exclusively liver‐specific (Fig. 1).

Regarding liver‐specific lncRNAs there are also some examples regularly mentioned in the literature, which may confuse readers. For instance, *lncLSTR* (aka “Liver‐Specific Triglyceride Regulator”). This lncRNA indeed has exclusive expression in liver tissues, interacts with TAR DNA‐binding protein 43 and interferes suppression of member of the cytochrome P450 Cyp8b1—a key enzyme in bile acid synthesis. Depletion of *lncLSTR* enhances ApoC2 expression through FXR‐mediated pathway and leads to activation of lipoprotein lipase and increase of plasma triglyceride levels [137]. However, all these data were obtained for mice only, and no human homolog was found for *lncLSTR* (NR_038011.1). Another example is *lnc‐HC* (aka “lncRNA derived from HepatoCytes”) that was differentially expressed in liver tissues of rat NAFLD model. *Lnc‐HC* (NR_148404.1) is a liver‐specific 1410 nt transcript, that localizes in nucleus and regulates Cyp7a1 and Abca1 expression through direct binding to hnRNPA2B1, thus controlling hepatic fatty acid and triglyceride metabolism [138]. *Lnc‐HC* negatively regulates PPARγ expression at the post‐transcriptional level through binding to co‐mediator miR‐130b‐3p [138]. Although *lnc‐HC* is often mentioned in the literature as NAFLD‐associated liver‐specific lncRNA, we did not find any evidence for its putative human homolog. Similar discrepancy might be found in some reviews describing murine‐specific lncRNA *LeXiS* (aka “Liver‐expressed LXR‐induced Sequence”), which is not initially liver‐specific in normal tissues, but is incredibly inducible in mouse primary hepatocytes upon treatment with GW3965—a synthetic agonist of liver X receptor (LXR) [139]. There is one similar novel lncRNA in humans also known for regulating hepatic lipid metabolism—*lncHR1* (aka “HCV regulated 1”, *RP11‐778 J16.3*/ ENSG00000257400). *LncHR1* was shown to repress *SREBP‐1c* gene expression [140]; however, *lncHR1* is not liver‐specific and has very low background expression in all human tissues and cancers.

## Conclusions

Current fast developing technologies for RNA sequencing and data analysis opened wide perspectives for identification of unique biomarkers specific for distinct types of tumors, including those, which might be secreted in biological fluids allowing noninvasive detection. Analysis of circulating tumor‐related exosomes might become a routine procedure in future and a lot of scientific efforts are focused on a search for appropriate cancer‐associated biomarkers, including lncRNAs. Numerous studies already have shown that exosomal lncRNAs might be applicable for HCC diagnostics, and dozens of such biomarkers circulating in patient's serum are described [141, 142]. However, none of them are HCC‐specific or liver‐specific; therefore, currently they may be considered only in terms of general oncomarker screening. We addressed our interest to the issue, how many liver‐specific lncRNAs are known, and whether some of them might be used as cancer biomarkers. In the present review we performed comprehensive literature search combined with validation of the data in the available databases, and highlighted all current findings in the field of liver‐specific lncRNAs. This resulted into a final list of known liver‐specific lncRNAs, which general features are listed in Table 1. Based on our work, we conclude that some lncRNAs initially claimed as liver‐specific cannot be undoubtedly considered as unique features of normal or cancerous liver tissues (e.g., *HULC*, *DBH‐AS1, LINC1018, LINC01146*), as they were also described in terms of other cancers. We also realized, that most of known lncRNAs with abundant expression in the liver are downregulated upon carcinogenesis (Table 1), thus likely cannot be considered for diagnostics. On the other hand, most of them are involved in the control of proliferation showing strong oncosuppressing functions and association with favorable patient survival. These features might be useful for their application as valuable prognostic panels and even as novel therapeutical strategies for liver cancer treatment, including HBV‐ and or HCV‐mediated liver cancers. We would like to note exceptional prognostic value of *LINC01554* in terms of HCC and its strong contribution to oncosupressing mechanisms, which might be further considered for its application in cancer therapy [88, 89, 90, 91, 92, 93, 94]. We also appreciate recent findings discovered functions of *FAM99B*, as well as first attempts to apply liver‐targeted delivery based on GalNAc conjugates for liver cancer treatment using truncated *FAM99B* molecules [116]. For sure, most of the described findings need further investigations, both for understanding of exact mechanisms of lncRNA functioning, as well as for validation of diagnostic/prognostic value on larger sample size of patients. However, we believe our review will shed the first light on the current state of knowledge in this field, and the summarized data will help future researchers.

**Table 1:** Known liver‐specific^a^ lncRNAs associated with liver cancers and their functions found in *in vitro* and/or *in vivo* experiments in cellular and/or murine models related to HCC.

LncRNA	Synonyms	Ensembl ID	Expression in HCC	Function^b^	Direct targets	Downstream targets/pathways	References
*HULC* ^a^	*LINC00078*	ENSG00000285219, previously ENSG00000251164	Up	Oncogene	Sponging miRs: miR‐134‐5p, miR‐383‐5p, miR‐377‐5p, miR‐3200‐5p, miR‐186, miR‐200a‐3p, miR‐372, miR‐15a, miR‐107 protein partners: YB‐1, LDHA, PKM2, HBXIP	Activation of FOXM1, VAMP2, HIF‐1α, ATF4, HMGA2, ZEB1, Prkacb/CREB, PTEN/Akt/Pi‐3K/mTOR, E2F1/SPHK1, USP22, SIRT1, COX‐2, NF‐kB, APOBEC3B	[24, 25, 26, 27, 71, 72, 73, 74, 75, 76, 77, 78, 79, 80]
*DBH‐AS1* ^a^	*BPR, NCRNA00118*	ENSG00000225756	Contradictory	Oncogene	Sponges miR‐138	Activation of phosphorylation levels of proteins of FAK/Src/ERK pathway	[81, 82, 83]
*LINC01554*	*C5orf27, FIS*	ENSG00000236882	Down	Oncosuppressor	Sponges miR‐3681‐3p, miR‐148b‐3p	Inhibition of NGFR, PI3K/AKT/mTOR and WNT/β‐catenin pathways Activation of EIF4E3, BAX, BCL2/BAX, p53, cleaved‐Caspase3	[88, 89, 90, 91, 92, 93, 94]
*LINC01018* ^a^	*SRHC*, *CTD‐2195M18.1*	ENSG00000250056	Down	Oncosuppressor	Sponges miR‐182‐5p, miR‐197‐3p	Activation of FOXO1, BAX, GNA14	[95, 96, 97, 98]
*LINC01348*	*HSALNG0142454*	ENSG00000280587	Down	Oncosuppressor	Binds SF3B3 and inhibit *EZH2* pre‐mRNA splicing	Inhibition of EZH2 and JNK/c‐Jun/SNAI1 pathway	[102]
*LINC02362* ^a^	*RP11‐290F5.1*	ENSG00000249096	Down	Oncosuppressor	Sponges miR‐516b‐5p, miR‐18a‐5p	Promotion of SOCS2 and FDX1 expression	[104, 105]
*LINC01146* ^a^	*HISLA, HIF1A*	ENSG00000258867	Down	Oncosuppressor	Unknown	Unknown, putatively co‐expressed with FETUB and TTR	[107, 108]
*Lnc‐DAW* ^a^	*LINC01620*, *LINC01430*	ENSG00000168746, previously ENSG00000237907	Contradictory	Oncogene	Binds EZH2 and causes its degradation	Activation of WNT/β‐catenin pathway	[109]
*FAM99A*	*FLJ42833*	ENSG00000281261, previously ENSG00000205866	Down	Oncosuppressor	Sponges miR‐299‐5p binds PCBP1, SRSF5, SRSF6, YBX1, IGF2BP2, HNRNPK HNRNPL	Inhibition of GLUT1, JAK2/STAT3 pathway	[111, 112, 113]
*FAM99B*	*NONHSAG007385.2*	ENSG00000281783, previously ENSG00000205865	Down	Oncosuppressor	Binds DDX21	Promotes DDX21 translocation of cytoplasm and further degradation; affects translation machinery via regulation of processing of pre‐rRNAs and DDX21‐mediated expression of RPS29 and RPL38	[105, 106, 107, 108, 109, 110, 111, 112, 113, 114, 115, 116]
*LINC01093*	*NONHSAG039504.2*	ENSG00000249173	Down	Oncosuppressor	Binds IGF2BP1 and promotes degradation of *GLI1* mRNA binds SIRT1	Down‐regulation of EGFR, FOXM1, VEGF and BCL‐XL Inhibition of ICAM‐1‐mediated NF‐κB pathway	[78, 117, 118, 119, 120]
*LINC02428*	*RP11‐328K4.1*	ENSG00000248740	Down	Oncosuppressor	Binds IGF2BP1	Degradation of *KDM5B* mRNA, repression of EMT	[121]
*LIVAR*	*RP11‐484N16.1, lnc18q22.2*	ENSG00000266304	Contradictory	Oncogene	Unknown	Activation of PARP1	[123]
*CPS1‐IT1*	*CPS1‐IT, CPS1IT, PRO0132*	ENSG00000280837	Down	Oncosuppressor	Binds Hsp90	Inactivation of HIF‐1α, repression of EMT	[125, 126, 127]
*LINC01702*	*RP4‐763G1.2*	ENSG00000235200	Down	No data	Unknown	Unknown	[123]
*SMUG1P1*	*NONHSAT059247.2*	ENSG00000267444	Down	No data	Unknown	Unknown	[129]
*LINC02499*	*RP11‐622A1.2*	ENSG00000250436	Down	Oncosuppressor	Unknown	Unknown	[131, 132, 133, 134]
*AC115619.1*	*NONHSAG027174.2, RP11‐116D2.1*	ENSG00000261012	Down	Oncosuppressor	Unknown	Unknown	[122, 135]
*HELIS*	*AC068535.3, LINC01831*	ENSG00000225765	Down	No data	Unknown	Unknown	[78]
*AC008549.1*	*LOC124901048*, *Lnc‐AP3S1‐2*, *CTC‐505O3.2*	ENSG00000248709	Down	No data	Unknown	Unknown	[136]

^a^ lncRNAs which cannot be undoubtedly considered as liver‐specific lncRNAs

^b^ based on *in vitro* and/or *in vivo* studies performing for knockdown and/or overexpression experiments on HCC cell model.

## Conflict of interest

The authors declare no conflict of interest.

## Peer review

The peer review history for this article is available at https://www.webofscience.com/api/gateway/wos/peer‐review/10.1002/2211‐5463.70079.

## Author contributions

OYB conceptualized the study; OYB and RKM prepared the original draft of the manuscript; MPR and OAD reviewed and edited the manuscript; OAD supervised the study; MPR acquired the funding for this study. All authors have read and agreed to the published version of the manuscript.

## Supporting information


**Fig. S1.** Expression levels [log_2_(TPM+1)] of lncRNAs in TCGA datasets for HCC and CCA tumors in comparison to TCGA normal samples visualized by GEPIA (gepia.cancer‐pku.cn).
**Fig. S2.** Overall survival of HCC patients with high/low expression of different lncRNAs according to the data from TCGA dataset for HCC (LIHC, Liver hepatocellular carcinoma) visualized by GEPIA (gepia.cancer‐pku.cn).
**Fig. S3.** Median gene‐level expression [log_2_(TPM+1)] of described lncRNAs across different tumors (TCGA datasets) visualized by GEPIA (gepia.cancer‐pku.cn) in a mode of Multiple Gene Comparison.
**Fig. S4.** Expression of different *LINC01620* transcripts in normal human tissues (GTEx data) visualized by FLIBase database (www.flibase.org).
