# Supplementary material for: Liver‐specific lncRNAs associated with liver cancers
Source: FEBS Open Bio. 2025 Jul 1;15(9):1383–405. doi: 10.1002/2211-5463.70079 (PMC12401184; doi:10.1002/2211-5463.70079)
Supplement: Supplementary file 1 — Fig. S1. Expression levels [log2(TPM+1)] of lncRNAs in TCGA datasets for HCC and CCA tumors in comparison to TCGA normal samples visualized by GEPIA (gepia.cancer‐pku.cn). Fig. S2. Overall survival of HCC patients with high/low expression of different lncRNAs according to the data from TCGA dataset for HCC (LIHC, Liver hepatocellular carcinoma) visualized by GEPIA (gepia.cancer‐pku.cn). Fig. S3. Median gene‐level expression [log2(TPM+1)] of described lncRNAs across different tumors (TCGA datasets) visualized by GEPIA (gepia.cancer‐pku.cn) in a mode of Multiple Gene Comparison. Fig. S4. Expression of different LINC01620 transcripts in normal human tissues (GTEx data) visualized by FLIBase database (www.flibase.org). [file FEB4-15-1383-s001.pdf]

Supplementary

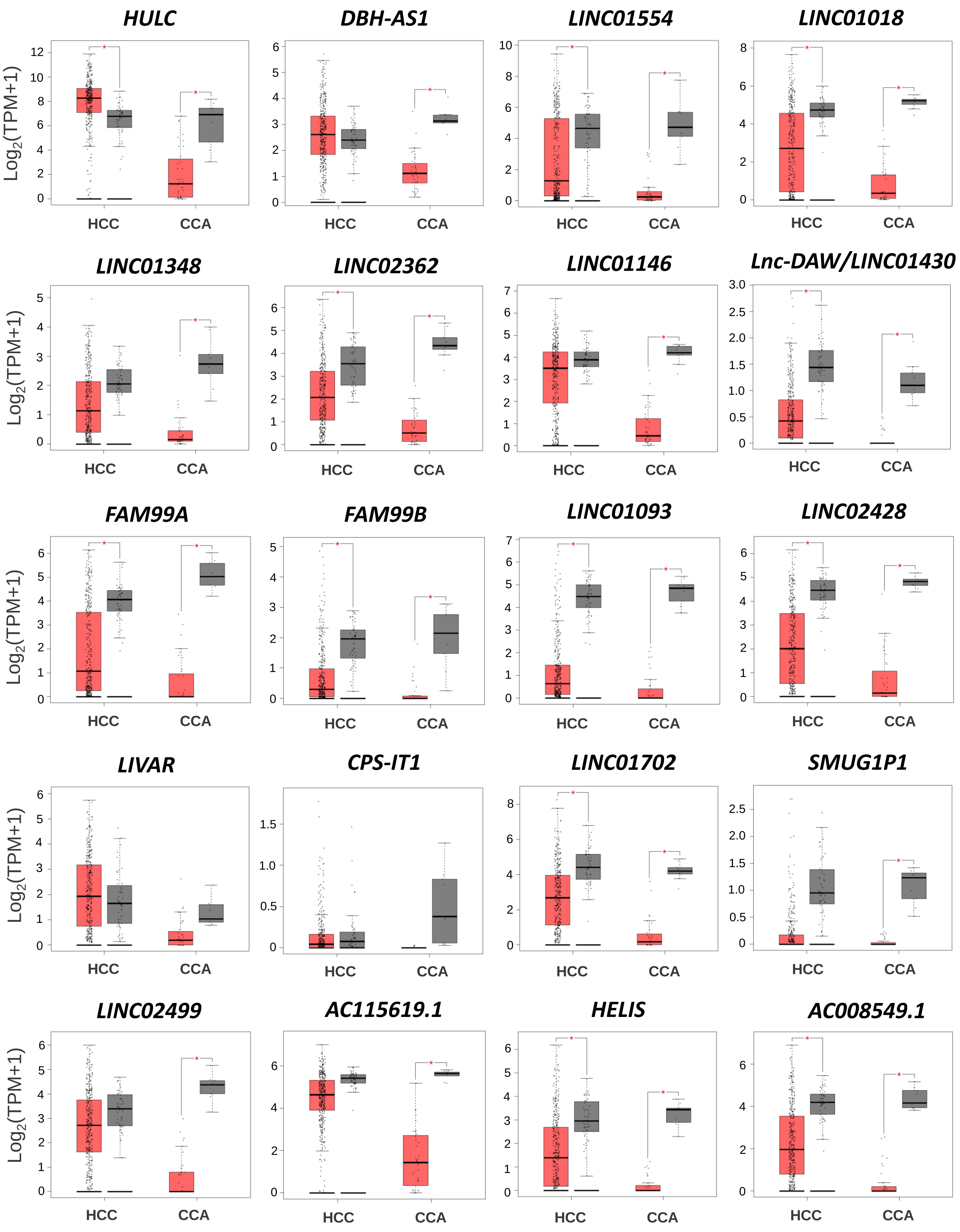

Figure S1. Expression levels [$\log_2$(TPM+1)] of lncRNAs in TCGA [1] datasets for hepatocellular carcinoma (HCC) and cholangiocarcinoma (CCA) tumors in comparison to TCGA normal samples [HCC(T)=369, HCC(N)=50; CCA(T)=36, CCA(N)=9] visualized by GEPIA (www.gepia.cancer-pku.cn, [2]).

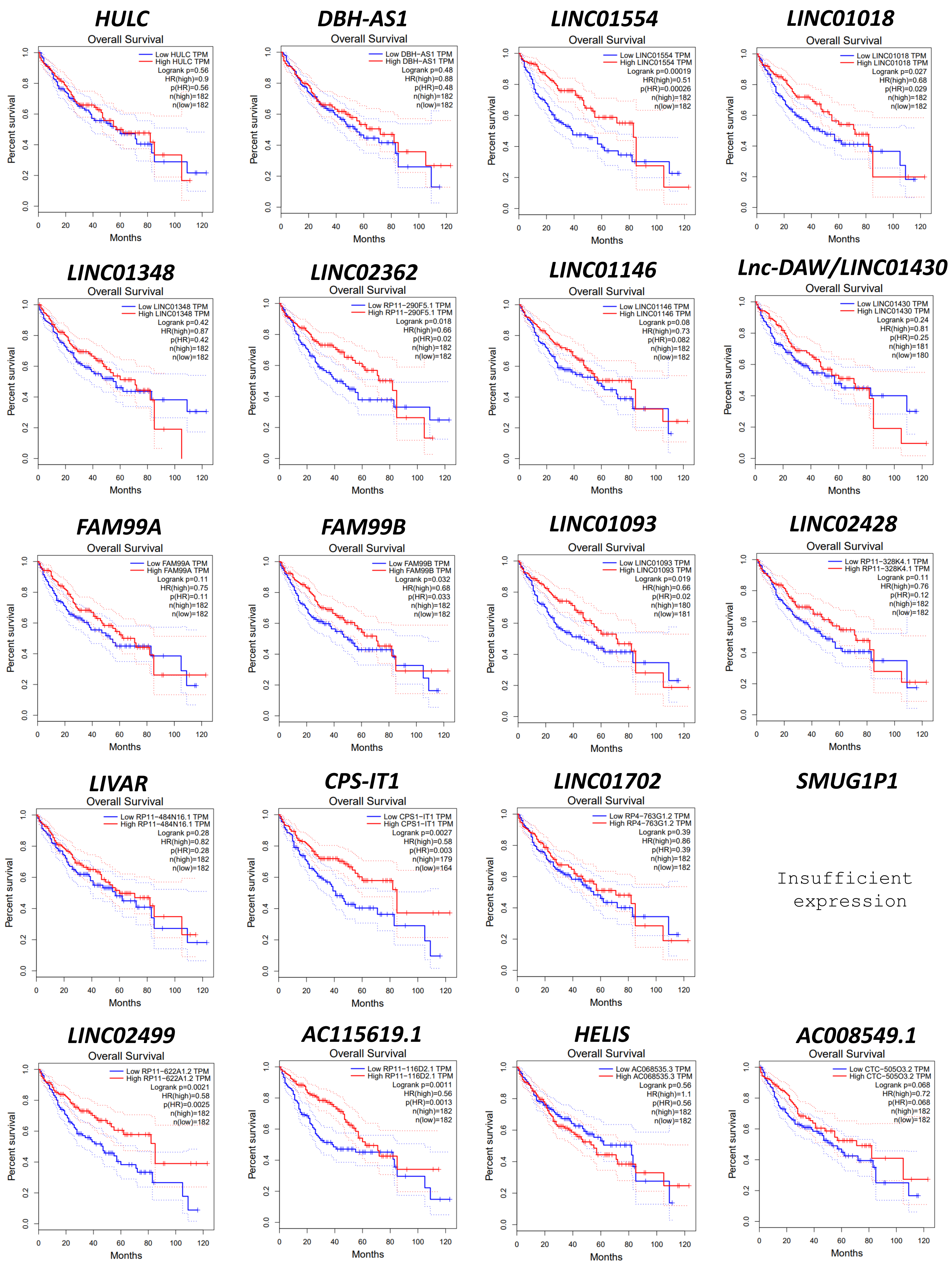

Figure S2. Overall survival of HCC patients with high/low expression of different lncRNAs according to the data from TCGA [1] dataset for HCC (LIHC, Liver hepatocellular carcinoma) visualized by GEPIA (www.gepia.cancer-pku.cn, [2]).

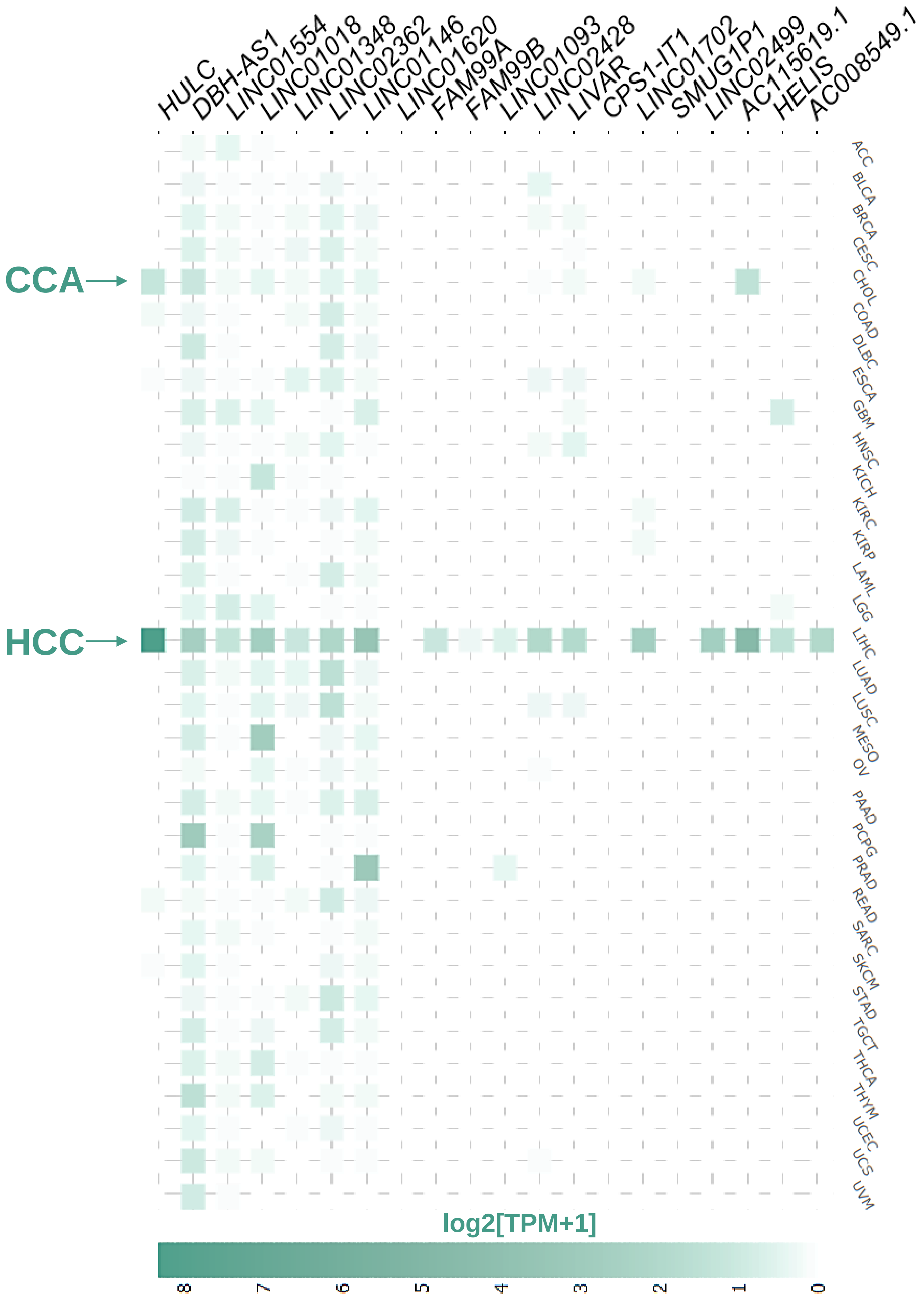

Figure S3. Median gene-level expression [log2(TPM+1)] of described lncRNAs across different tumors (TCGA [1] datasets) visualized by GEPIA (www.gepia.cancer-pku.cn, [2]) in a mode of Multiple Gene Comparison. Abbreviations: ACC: Adrenocortical carcinoma; BLCA: Bladder urothelial carcinoma; BRCA: Breast invasive carcinoma; CESC: Cervical squamous cell carcinoma and endocervical adenocarcinoma; CHOL: Cholangiocarcinoma; COAD: Colon adenocarcinoma; DLBC: Lymphoid neoplasm diffuse large B cell lymphoma; ESCA: Esophageal carcinoma; GBM: Glioblastoma multiforme; HNSC: Head and neck squamous cell carcinoma; KICH: Kidney chromophobe; KIRC: Kidney renal clear cell carcinoma; KIRP: Kidney renal papillary cell carcinoma; LAML: Acute myeloid leukemia; LGG: Brain lower grade glioma; LIHC: Liver hepatocellular carcinoma; LUAD: Lung adenocarcinoma; LUSC: Lung squamous cell carcinoma; MESO: Mesothelioma; OV: Ovarian serous cystadenocarcinoma; PAAD: Pancreatic adenocarcinoma; PCPG: Pheochromocytoma and paraganglioma; PRAD: Prostate adenocarcinoma; READ: Rectum adenocarcinoma; SARC: Sarcoma; SKCM: Skin cutaneous melanoma; STAD: Stomach adenocarcinoma; TGCT: Testicular germ cell tumors; THCA: Thyroid carcinoma; THYM: Thymoma; UCEC: Uterine corpus endometrial carcinoma; UCS: Uterine carcinosarcoma; UVM: Uveal melanoma.

Table of transcripts:

TranscriptID	Gene Symbol	Length	Exons
TCONS_00661961 (ENST00000637248.1)	LINC01620	546	3
TCONS_00661962	LINC01620	1245	4
TCONS_00661963	LINC01620	1130	5
TCONS_00661964 (ENST00000415299.2)	LINC01620	452	3
TCONS_00661965 (ENST00000372910.5)	LINC01620	649	4
TCONS_00661966 (ENST00000306731.4)	LINC01620	466	3
TCONS_00661967 (ENST00000623295.1)	LINC01620	700	2

Gene model:

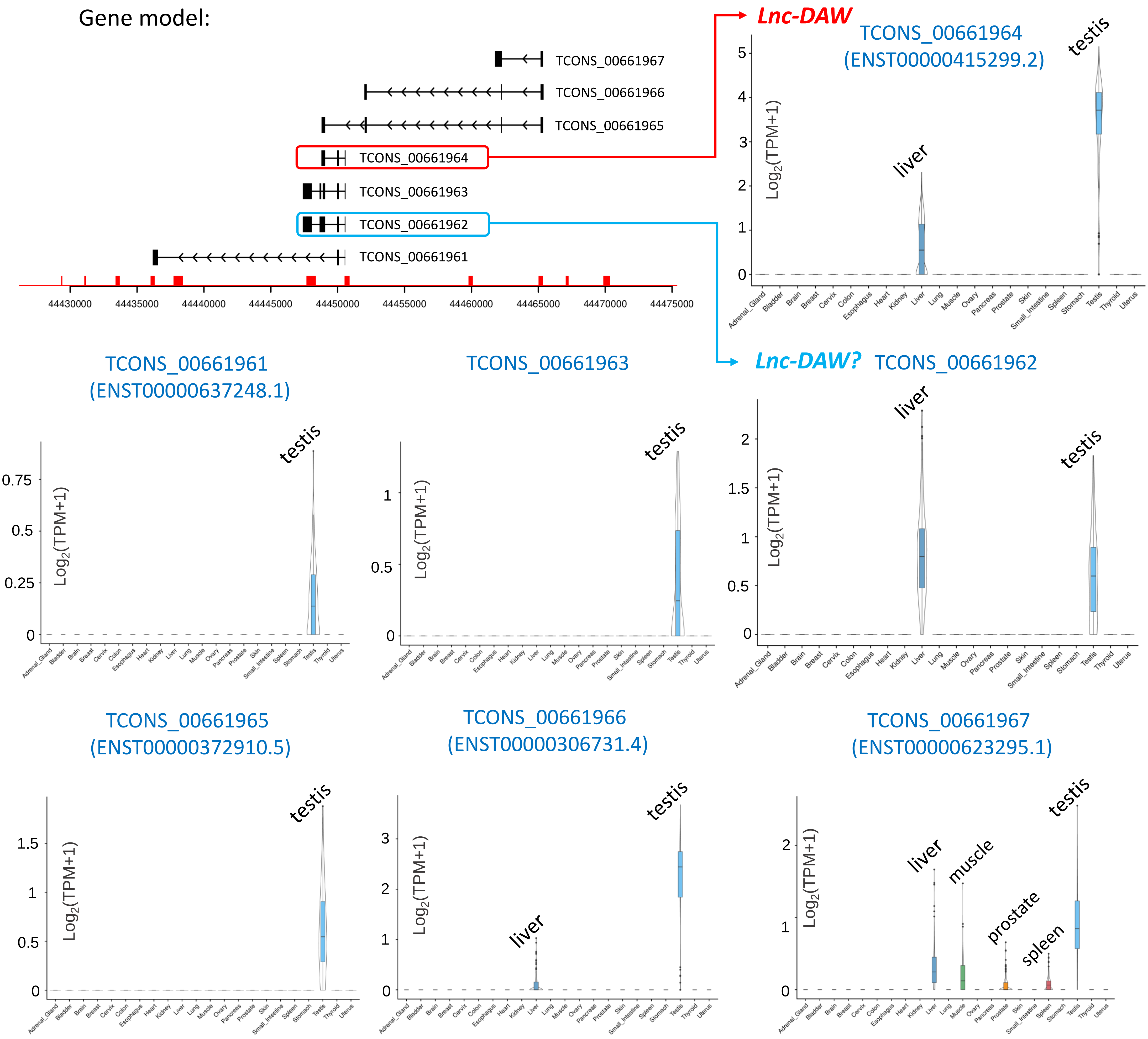

Figure S4. Expression of different *LINC01620* transcripts in normal human tissues (GTEx data) visualized by FLIBase database (www.flibase.org, [3]).

References

1. Cancer Genome Atlas Research Network; Weinstein JN, Collisson EA, Mills GB, Shaw KR, Ozenberger BA, Ellrott K, Shmulevich I, Sander C, Stuart JM. (2013) The Cancer Genome Atlas Pan-Cancer analysis project. *Nat Genet* 45:1113–1120. doi: 10.1038/ng.2764
2. Tang Z, Li C, Kang B, Gao G, Li C, Zhang Z. (2017) GEPIA: a web server for cancer and normal gene expression profiling and interactive analyses. *Nucleic Acids Res* 45: W98-W102. doi: 10.1093/nar/gkx247
3. Shi Q, Li X, Liu Y, Chen Z, He X. (2024) FLIBase: a comprehensive repository of full-length isoforms across human cancers and tissues. *Nucleic Acids Res*. 52: D124-D133. doi: 10.1093/nar/gkad745
